# Central Centrifugal Cicatricial Alopecia: A Survey of Treatment Practices Among Dermatology Residents and Attending Physicians

**DOI:** 10.7759/cureus.92780

**Published:** 2025-09-20

**Authors:** Janet Choi, Isabelle Ilan, Jin Ning Tian, Eliza Balazic, Christy Nwankwo, Kseniya Kobets

**Affiliations:** 1 Division of Dermatology, Albert Einstein College of Medicine, Bronx, USA; 2 Ronald O. Perelman Department of Dermatology, New York University (NYU) Grossman School of Medicine, New York, USA

**Keywords:** central centrifugal cicatricial alopecia, hair disorders, scalp inflammation, scarring alopecia, survey

## Abstract

Introduction

Central centrifugal cicatricial alopecia (CCCA) is a type of primary scarring alopecia characterized by hair loss on the vertex of the scalp. While treatment often focuses on reducing inflammation, no established standard of care exists. Early diagnosis and intervention are critical, as advanced stages with permanent follicular scarring are challenging to treat. Our study aims to assess CCCA treatment practices and provider confidence among dermatology residents and attendings in an urban academic setting. We further compare these real-world practices with existing literature.

Methods

We conducted a cross-sectional survey study of the commonly prescribed/recommended treatments for CCCA in 31 residents and attending physicians at two dermatology programs. Of those, 26 indicated that they see patients with CCCA, comprising 17 residents and nine attending physicians.

Results

Overall, the most frequently utilized therapies for CCCA were topical corticosteroids (TCS) (n = 26), designated as first-line by 90.5% of users, topical minoxidil (n = 24), used first-line by 77.3%, and intralesional corticosteroids (n = 24), used first-line by 65% of users. Oral tetracyclines were used by only 56% (n = 14) of respondents.

Discussion

Our findings mostly align with the current recommendations in the literature, focusing on reducing inflammation with topical and intralesional corticosteroids. Interestingly, topical minoxidil was used first-line by most respondents, possibly reflecting its greater accessibility to our patient population. The discrepancies in our findings may be reflective of variability in provider comfort in CCCA management, concern for side effects of systemic treatments, and presentations of later, “burnt-out” stages of CCCA.

## Introduction

Central centrifugal cicatricial alopecia (CCCA) is a type of scarring alopecia that predominantly affects middle-aged women of African descent, characterized by hair loss at the vertex of the scalp in a centrifugal pattern, and affects an estimated 15% of Black women [1,2]. The pathogenesis is not well understood. CCCA involves premature desquamation of the inner hair root sheath, leading to an inflammatory cascade and eventual destruction of the follicle [3]. On histopathology, CCCA is characterized by the absence of the inner root sheath and the presence of compound follicular structures with perifollicular fibrosis [4]. CCCA is thought to have a multifactorial etiology with both genetic and environmental components, such as, traction-inducing hairstyles, bacterial scalp infections, and diabetes mellitus type 2 [5,6]. Management typically focuses on reducing inflammation and halting the progression of the scarring process [7]. Early diagnosis and intervention are crucial, as advanced stages with clinical perifollicular fibrosis are challenging to treat as the pilosebaceous units become permanently scarred. However, patients have reported several barriers to effective care for CCCA, including limited physician experience with CCCA or in managing ethnic hair and limited access to effective treatment options [8]. Currently, there are no published randomized controlled trials on treatment for CCCA or established clinical guidelines for the management of CCCA [7]. In 2024, Jackson et al. published a Delphi consensus survey proposing several treatment recommendations for CCCA based on input from 21 dermatologists with expertise in hair and scalp disorders [9]. Despite growing recognition of CCCA, there remains a paucity of data on how it is typically managed in clinical practice. Our study aims to assess CCCA treatment practices and provider confidence among dermatology residents and attendings in an urban academic setting. We further compare these real-world practices with existing literature, including the 2024 Delphi consensus. To our knowledge, this is the first survey-based study to evaluate current CCCA treatment practices.

## Materials and methods

We conducted a cross-sectional, IRB-exempt survey study at the Albert Einstein College of Medicine/Montefiore Medical Center and St. Barnabas Hospital, located in New York. Albert Einstein College of Medicine Institutional Review Board issued approval 2022-14189. Prior to distribution, the questionnaire was peer-reviewed by two board-certified dermatologists. The questionnaire was distributed to all residents and attending physicians of the Dermatology Division/Department at both residency programs in October 2024 (Appendix 1). It was conducted online via a Qualtrics link for ease of access and distribution.

The survey consisted of 10 questions regarding the respondents (experience, confidence in treating CCCA, etc.) and up to 74 questions regarding CCCA treatment regimens, with conditional follow-up questions based on prior responses (Appendix 1). The questionnaire included a question asking to indicate the race, with response options: Asian, Black, Native Hawaiian or Other Pacific Islander, American Indian or Alaskan Native, and White participants. Treatment options specified in the survey reflected the most commonly used treatments for CCCA in the literature, including ketoconazole shampoo, ciclopirox shampoo, zinc pyrithione shampoo, selenium sulfide shampoo, topical corticosteroids, intralesional corticosteroids, topical minoxidil, oral minoxidil, oral finasteride, oral tetracyclines, light-emitting diode (LED) light therapy, platelet-rich-plasma (PRP), exosomes, compounded topical medications, and systemic corticosteroids [2,9]. Follow-up questions included, but were not limited to, the respondent’s likelihood of prescribing or recommending the treatment, confidence in its efficacy, and the concentration used. In addition to multiple-choice responses, respondents had an option to provide free-text comments. Data were summarized, and descriptive statistics were analyzed using Microsoft Excel, version 16.99.1 (Microsoft Corporation, Redmond, Washington, United States).

## Results

The survey was distributed to a total of 35 providers, with 31 initiating the survey, resulting in a response rate of 88.6%. Of those, 26 indicated that they see patients with CCCA, comprising 17 residents and nine attending physicians (Table 1). 

**Table 1:** Summary of patients seen by participants All data are reported as n (%). SOC: skin of color; CCCA: central centrifugal cicatricial alopecia

Parameters	Residents (n = 17)	Attendings (n = 9)	Combined (n = 26)
Percent of mix of SOC seen in your practice, n (%)			
25%	0 (0)	2 (22.2)	2 (7.7)
50%	3 (17.7)	3 (33.3)	6 (23.1)
75%	9 (52.9)	3 (33.3)	12 (46.1)
>75%	5 (29.4)	1 (11.1)	6 (23.1)
Number of CCCA patients seen per week, n (%)			
0	1 (5.9)	0 (0)	1 (3.8)
<3	15 (88.2)	7 (77.8)	22 (84.6)
4 to 10	1 (5.9)	1 (11.1)	2 (7.7)
11 to 20	0 (0)	1 (11.1)	1 (3.8
Percent of your patients willing to use out-of-pocket therapies, n (%)			
0%	2 (11.8)	1 (11.1)	3 (11.5)
<10%	7 (41.2)	3 (33.3)	10 (38.5)
25%	4 (23.5)	2 (22.2)	6 (23.1)
50%	5 (23.5)	2 (22.2)	7 (23.1)
75%	0 (0)	1 (11.1)	1 (3.8)
Comfort with treating hair loss, n (%)			
Very comfortable	1 (5.9)	3 (33.3)	4 (15.4)
Comfortable	7 (41.2)	4 (44.4)	11 (42.3)
Neutral	8 (41.2)	1 (11.1)	8 (30.8)
Uncomfortable	2 (11.8)	1 (11.1)	3 (11.5)
Comfort with treating CCCA, n (%)			
Very comfortable	0 (0)	2 (22.2)	2 (7.7)
Comfortable	4 (23.5)	4 (44.4)	8 (30.8)
Neutral	8 (47.1)	2 (22.2)	10 (38.5)
Uncomfortable	5 (29.4)	1 (11.1)	6 (23.1)

**Figure 1:**
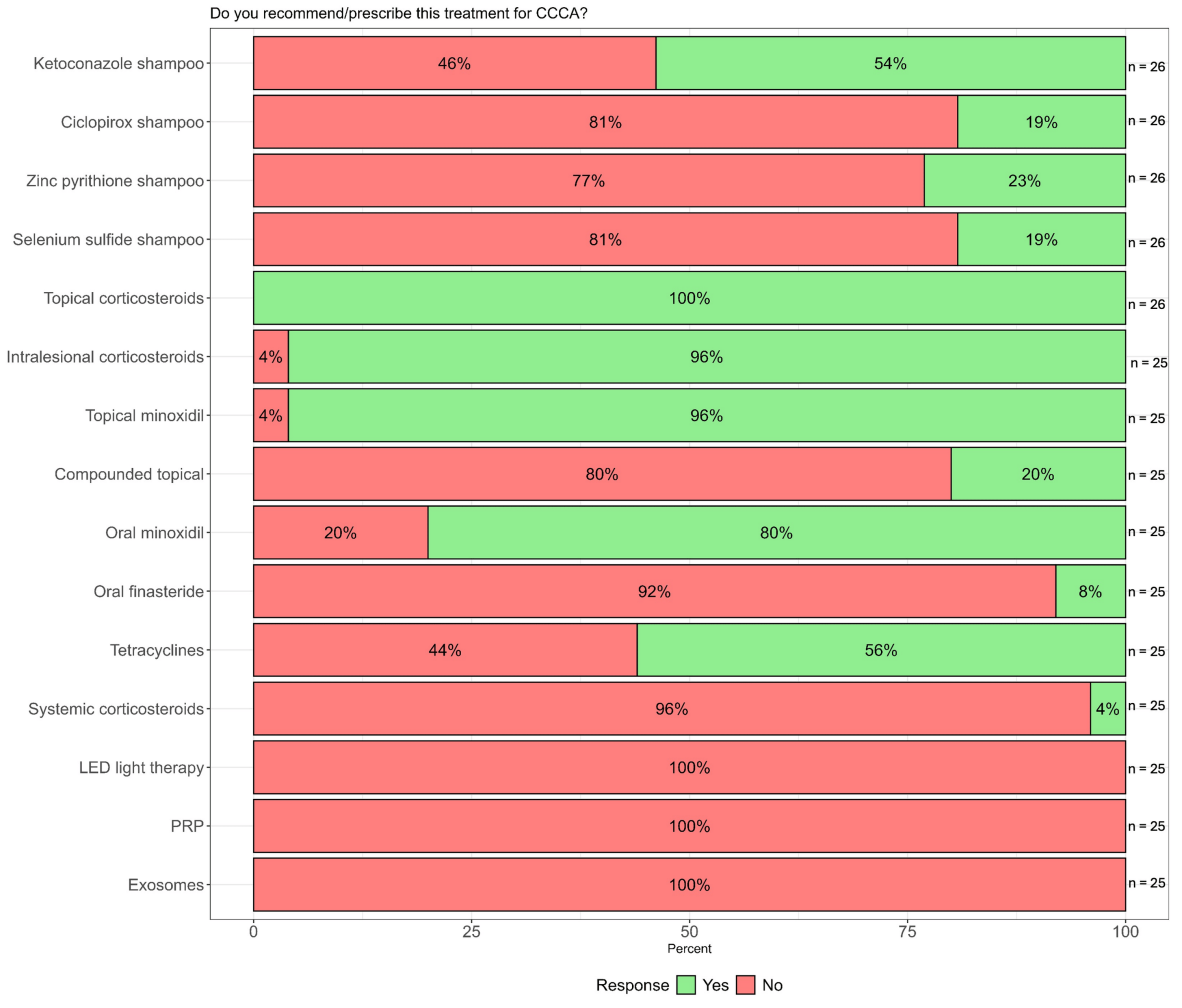
Summary of therapies for CCCA utilized by dermatology residents and attending physicians CCCA: central centrifugal cicatricial alopecia; LED: light-emitting diode; PRP: platelet-rich plasma; n: number of respondents

Most respondents reported seeing one to three CCCA patients per week (22/26, 84.6%), and the majority (24/26, 92.3%) indicated that at least 50% of their patient population were patients with skin of color (SOC). Most residents reported feeling neutral to uncomfortable with treating CCCA (13/17, 76.5%), while most attendings reported feeling comfortable to very comfortable with treating CCCA (6/9, 66.7%). Furthermore, 73.1% of respondents reported that the majority of their patients were unwilling to pursue out-of-pocket therapies. Figure 1 provides a summary of the CCCA treatments utilized by providers.

Medicated shampoos (ketoconazole, selenium sulfide, zinc pyrithione, ciclopirox)

Ketoconazole shampoo was the most frequently used (14/26, 53.8%), followed by zinc pyrithione (6/26, 23.1%), selenium sulfide (5/26, 19.2%), and ciclopirox shampoo (5/26, 19.2%) to treat CCCA. All respondents who reported using ciclopirox shampoo were residents (n = 5), with 60% (3/5) expressing a neutral level of confidence in its efficacy.

Other localized medications (topical corticosteroids, intralesional corticosteroids, topical minoxidil, compounded topical medications)

**Figure 2:**
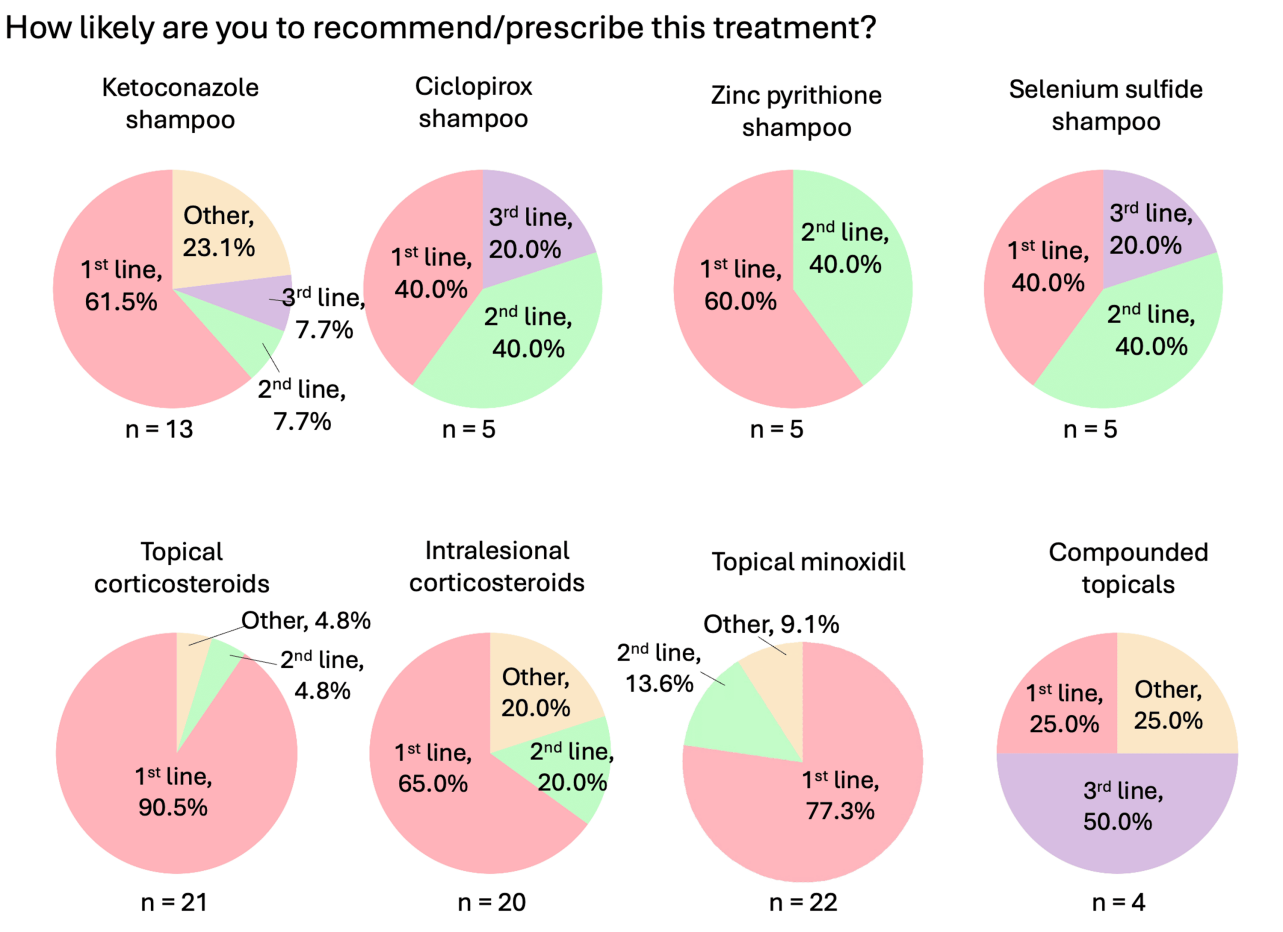
Localized therapies used for CCCA (general questions: likelihood of recommending/prescribing) Question was only asked if the respondent answered “yes” to recommending/prescribing the relevant therapy. “Other” responses include “with erythema and scale” for intralesional corticosteroids, and “adjunct” for topical minoxidil. CCCA: central centrifugal cicatricial alopecia; n: number of respondents

**Figure 3:**
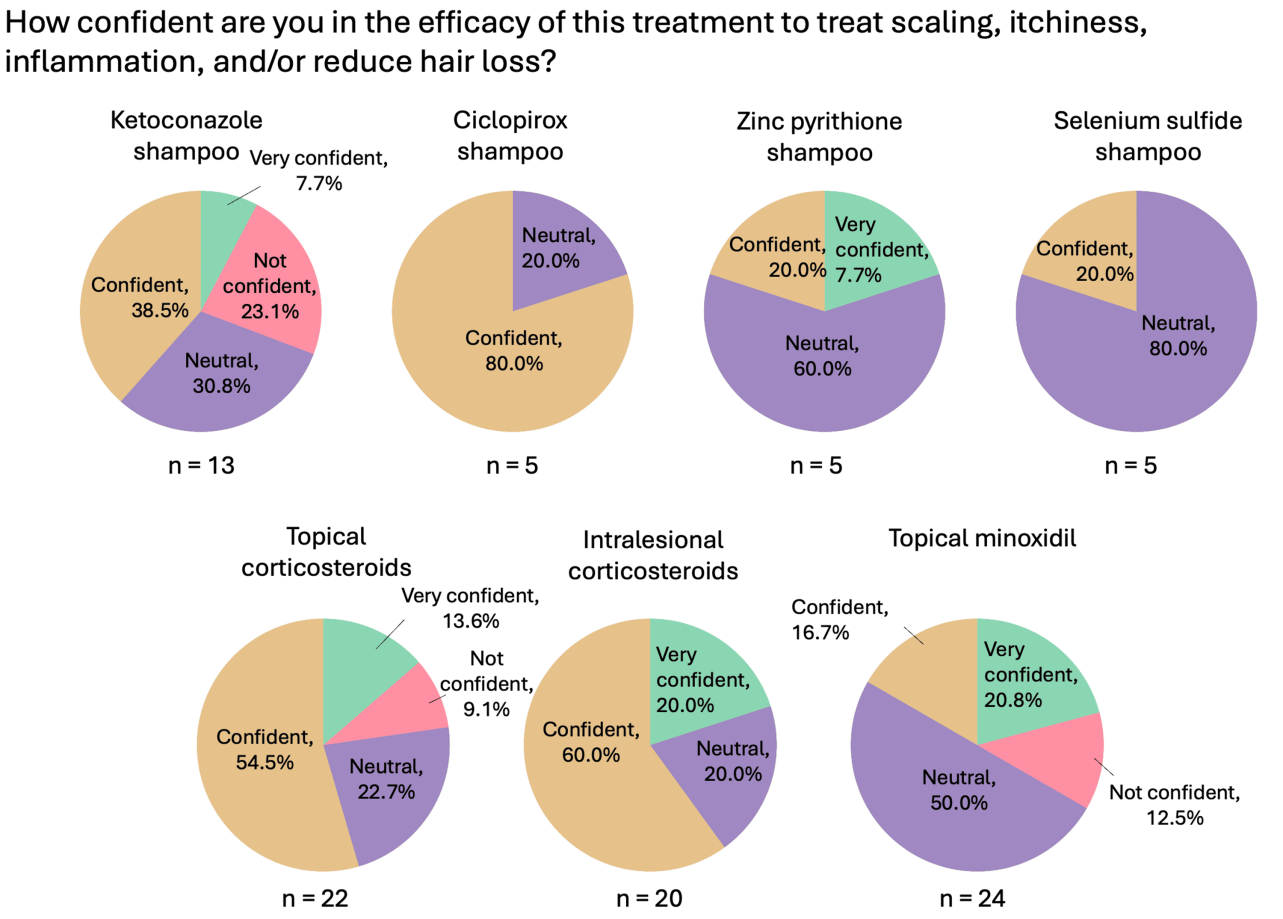
Localized therapies used for CCCA (general questions: confidence) Question was only asked if the respondent answered “yes” to recommending/prescribing the relevant therapy. CCCA: central centrifugal cicatricial alopecia; n: number of respondents

**Figure 4:**
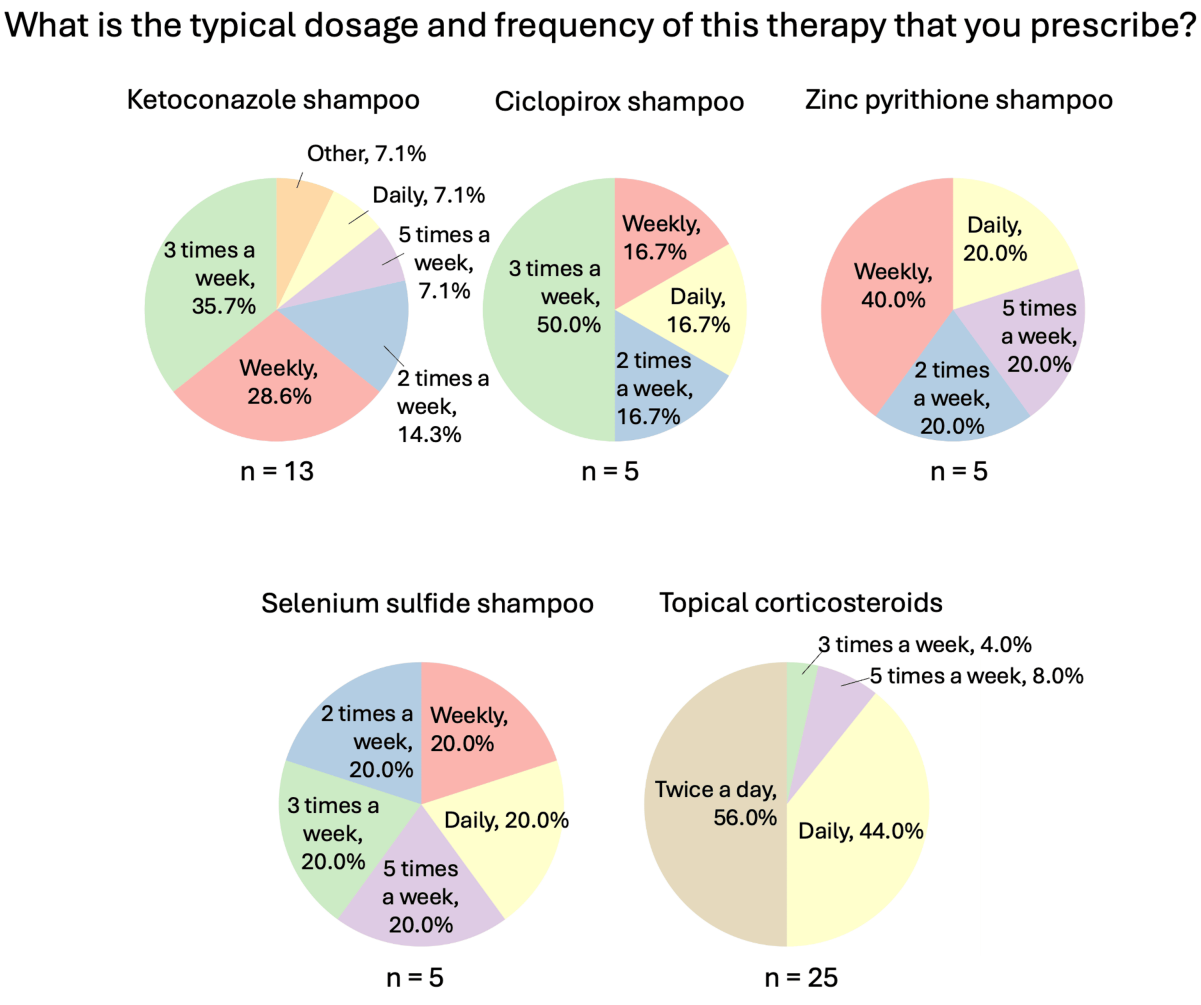
Localized therapies used for CCCA (general questions: dosage/frequency) Question was only asked if the respondent answered “yes” to recommending/prescribing the relevant therapy. Respondents could choose more than one answer, and therefore the sum of percentages may sum up to over 100%. CCCA: central centrifugal cicatricial alopecia; n: number of respondents

**Figure 5:**
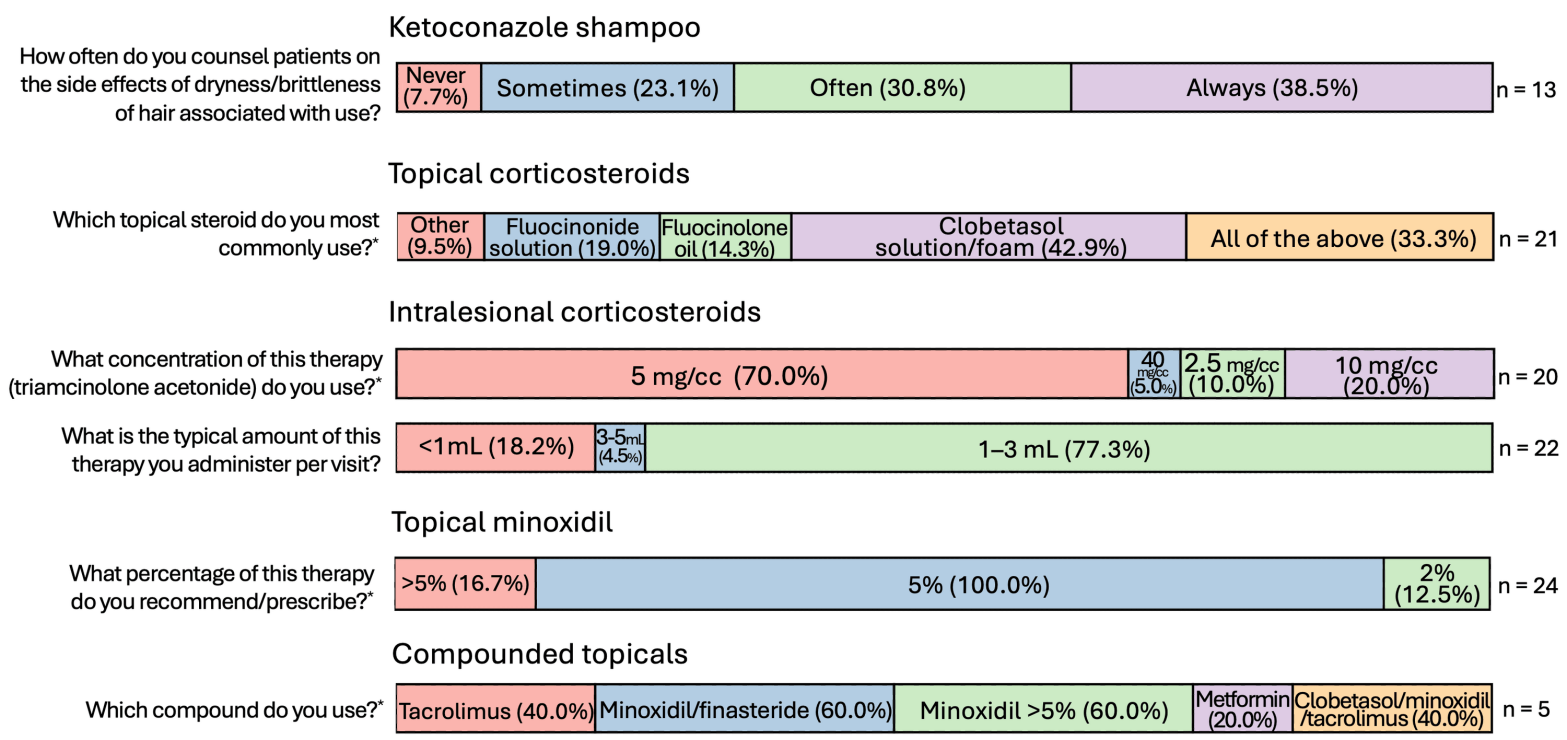
Localized therapies used for CCCA, therapy-specific questions Questions were only asked if the respondent answered “yes” to recommending/prescribing the relevant therapy. For the second question (“Which topical steroid do you most commonly use?”), “Other” responses include “betamethasone dipropionate gel” and “betamethasone dipropionate lotion.” *  denotes questions where respondents could choose more than one answer, and therefore the sum of percentages may sum up to over 100%. CCCA: central centrifugal cicatricial alopecia; n: number of respondents

Figures 2-5 summarize the responses to questions regarding localized therapies used for CCCA. All respondents (26/26, 100%) reported using topical corticosteroids (TCS) in their treatment of CCCA, with clobetasol solution/foam being the most common formulation (9/21, 42.9%) and twice-daily application as the most prescribed frequency (14/25, 56%). All but one respondent reported using intralesional corticosteroids (24/25, 96%) (Figure 1), most commonly as triamcinolone acetonide 5 mg/cc (14/20, 70%). Nearly all respondents reported using topical minoxidil 5% (24/25, 96%). Compounded topical medications were only used by 20% (5/25) of respondents, most frequently combining topical minoxidil and finasteride (3/5, 60%) or compounding topical minoxidil at high concentrations >5% (3/5, 60%).

Systemic medications (oral minoxidil, oral finasteride, oral tetracyclines, systemic corticosteroids)

**Figure 6:**
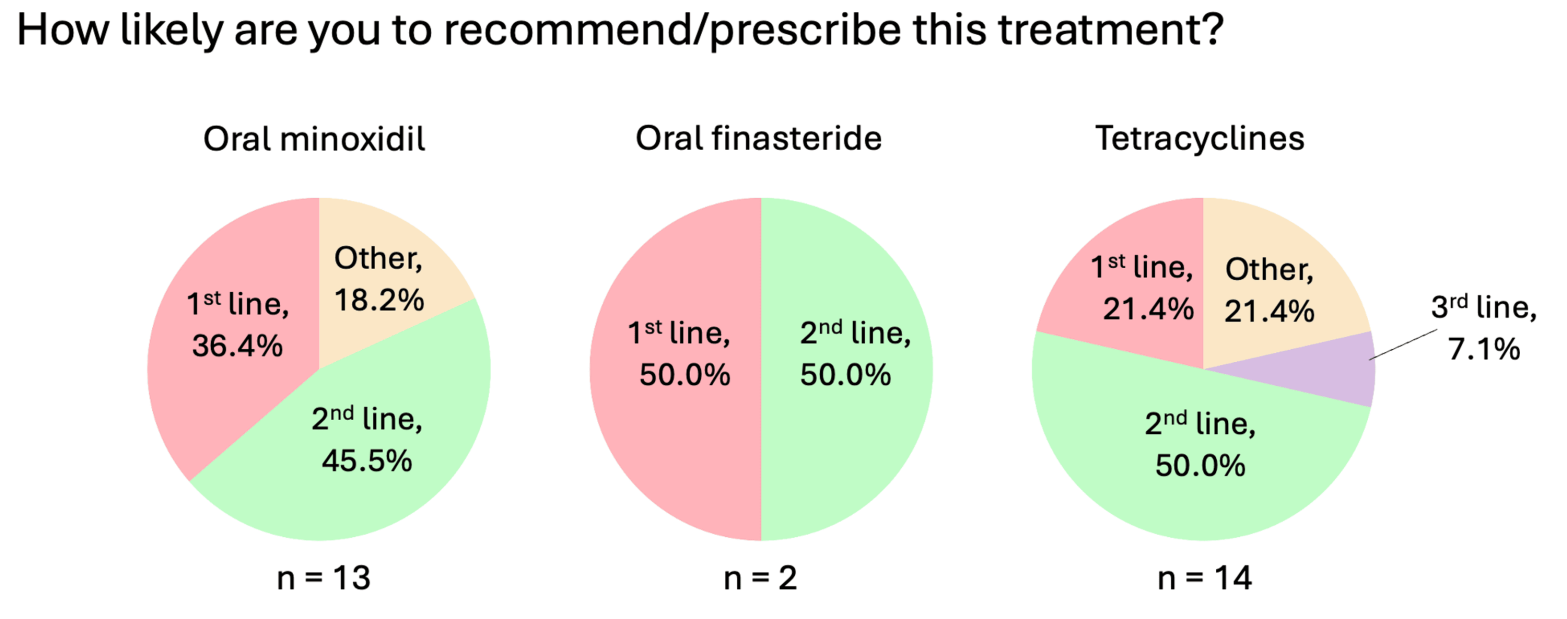
Systemic therapies used for CCCA (general questions: likelihood of recommending/prescribing) “Other” responses for oral minoxidil include “adjunct,” and for tetracyclines include “depends on the amount of inflammation, scarring, extent, patient's age, etc.” CCCA: central centrifugal cicatricial alopecia; n: number of respondents

**Figure 7:**
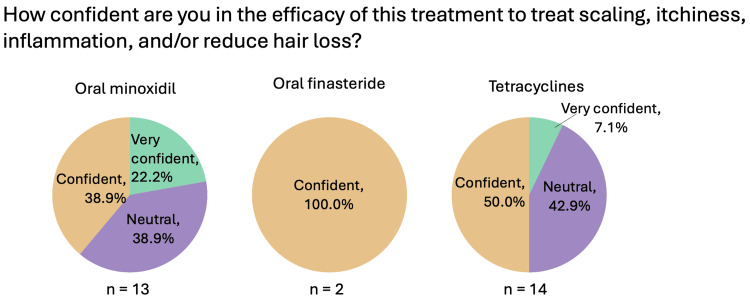
Systemic therapies used for CCCA (general questions: confidence) Question was only asked if the respondent answered “yes” to recommending/prescribing the relevant therapy. CCCA: central centrifugal cicatricial alopecia; n: number of respondents

**Figure 8:**
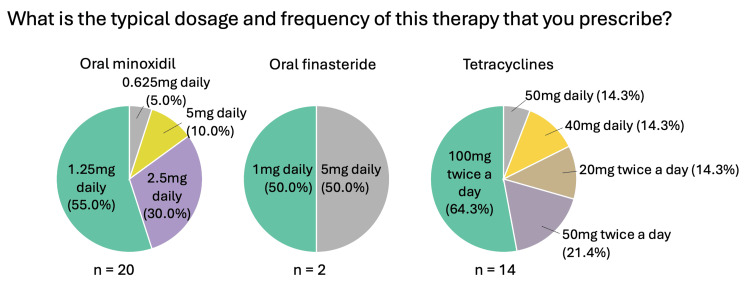
Systemic therapies used for CCCA (general questions: dosage/frequency) Question was only asked if the respondent answered “yes” to recommending/prescribing the relevant therapy. Respondents could choose more than one answer, and therefore, the sum of percentages may sum up to over 100%. CCCA: central centrifugal cicatricial alopecia; n: number of respondents

**Figure 9:**
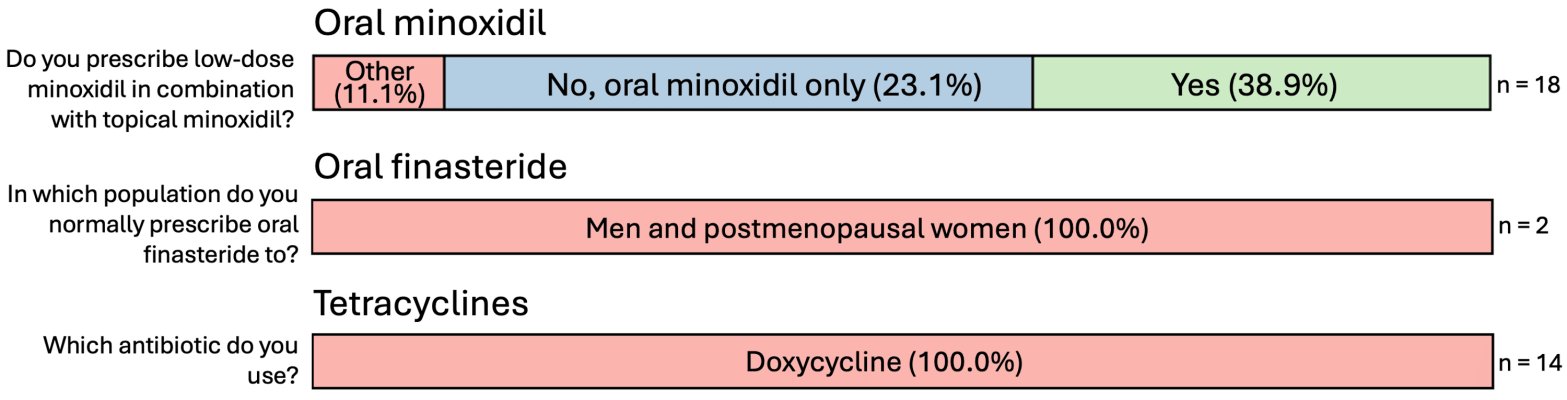
Systemic therapies used for CCCA, therapy-specific questions Questions were only asked if the respondent answered “yes” to recommending/prescribing the relevant therapy. For the first question (“Do you prescribe low-dose minoxidil in combination with topical minoxidil?”), “Other” responses for oral minoxidil include “patient dependent” and “sometimes.” CCCA: central centrifugal cicatricial alopecia; n: number of respondents

Figures 6-9 summarize the responses to questions regarding systemic therapies used for CCCA. Most respondents reported using oral minoxidil (20/25, 80%), although those who did commonly recommended this treatment as second-line (5/11, 45.5%). Oral finasteride was prescribed by only 8% (2/25), all in post-menopausal women. Oral tetracyclines, specifically doxycycline, were used by 56% (14/25), and of those, most used them as second-line therapy (7/14, 50%). The most common dosage was 100 mg twice daily (9/14, 64.3%). Systemic corticosteroids were rarely used (1/25, 4%), consisting of a single resident (Figure 1).

Miscellaneous treatments (in-office LED light therapy, platelet-rich plasma, exosomes)

None of the respondents reported using LED light therapy, PRP, or exosomes for CCCA management. Overall, the most frequently utilized therapies for CCCA were topical corticosteroids (n = 26), designated as first-line by 90.5% of users (19/21), topical minoxidil (n = 24), used first-line by 77.3% (16/24), and intralesional corticosteroids (n = 24), used first-line by 65% (13/20) of users.

## Discussion

This study captures real-world clinical approaches to the management of CCCA at two academic institutions and highlights variations in treatment preferences and confidence levels among residents and attending dermatologists.

Topical and intralesional corticosteroids are the mainstays of CCCA therapy, used to reduce inflammation and perifollicular edema of the scalp and to relieve symptoms including pruritus, tenderness, erythema, and scaling [10]. Nearly all respondents reported using either modality, and both were frequently designated as first-line therapies. Among the topical corticosteroids, clobetasol solution/foam was the most commonly prescribed type. For intralesional injections, 5 mg/cc of triamcinolone acetonide was the most commonly used concentration. These practices align with the Delphi consensus recommendations and the current literature, which emphasize that first-line therapy should primarily target inflammation to preserve the remaining hair [9,11].

Medicated antiseborrheic shampoos were utilized less frequently, with the most common being ketoconazole shampoo (n = 14). Although primarily known for its role as an antifungal agent, ketoconazole has also been shown to exhibit anti-inflammatory properties via inhibition of 5-lipoxygenase, supporting its use in non-fungal inflammatory scalp conditions [12]. Of the antiseborrheic shampoos, zinc pyrithione and ciclopirox-based shampoos have been suggested as better tolerated in African American women who chemically straighten their hair, as other formulations may contribute to dryness and hair breakage [13]. Comparatively, the dermatologists in the Delphi consensus by Jackson et al. did not reach agreement on the use of medicated shampoos [9].

Topical (n = 24) and oral minoxidil (n = 20) were widely utilized by respondents, reflecting provider efforts to promote hair growth alongside anti-inflammatory strategies [11]. Minoxidil, a vasodilator, has been shown to stimulate hair growth by shortening the telogen phase, prolonging the anagen phase, and increasing hair follicle size [14]. The Delphi consensus strongly supported the use of both topical and oral minoxidil as adjunctive therapies [9]. In contrast to these recommendations, most respondents in our study indicated using topical minoxidil as first-line therapy. In alignment with the consensus, oral minoxidil was more commonly used as a second-line or adjunctive therapy. We speculate that the more frequent use of topical minoxidil as a first-line therapy may be reflective of its over-the-counter availability and greater accessibility to our patient population. Although the efficacy of minoxidil in CCCA has not yet been established in randomized trials, its proposed benefit is thought to be due to stimulating intact follicles that have not yet been scarred and potentially treating a component of overlapping androgenetic alopecia (AGA) [15]. Histopathologic examinations of CCCA have shown the presence of follicular miniaturization, supporting the rationale for minoxidil use [4].

Compounded topical medications allow for the creation of formulations that are not typically found in commercially available products. Compounded topicals were only used by a small minority of the respondents (n = 5). The formulations reported to be prescribed by respondents were tacrolimus, minoxidil combined with finasteride, high-concentration minoxidil (>5%), metformin, and combined clobetasol, minoxidil, and tacrolimus. Metformin, an antidiabetic medication used for glycemic control, has been shown in case studies to promote hair growth in CCCA when applied topically [16]. Its proposed mechanism includes reducing circulating androgen activity and fibrosis through the mediation of adenosine monophosphate-activated protein kinase. Meanwhile, topical calcineurin inhibitors in CCCA may be beneficial in symptom alleviation from their anti-inflammatory effects by suppressing the synthesis of pro-inflammatory cytokines [17]. Overall, data on the efficacy of compounded topicals for CCCA are limited, and no specific recommendations for these medications reached agreement in the Delphi consensus [9].

Oral tetracyclines, specifically doxycycline, were reported to be used by approximately half of the respondents (n = 14), most commonly at a dosage of 100 mg twice daily. This dosing aligns with the Delphi consensus, which reached strong agreement on the use of doxycycline 200 mg daily for up to six months in adults with active CCCA [7]. Tetracyclines are bacteriostatic antimicrobial agents with anti-inflammatory effects, making them a therapeutic option in inflammatory scalp conditions [18]. Doxycycline use in CCCA has been supported by a 2024 retrospective study by Obi et al., which showed that patients who were given low-dose doxycycline in the initial management of CCCA in addition to topical/intralesional corticosteroids experienced greater hair regrowth compared to those who did not receive doxycycline [19]. We propose that the lower utilization of oral tetracyclines by the respondents may reflect hesitancy due to their frequent gastrointestinal side effects, such as nausea and diarrhea. Alternatively, this finding may indicate that some providers are seeing patients in later, “burnt-out” stages of CCCA where active inflammation is minimal.

In contrast, other systemic therapies, including oral finasteride (n = 2) and corticosteroids (n = 1), were sparsely used, which we postulate may be due to reluctance in prescription due to less evidence substantiating their use in CCCA and the risk of side effects. Finasteride, a 5α-reductase inhibitor, is a common treatment for AGA, and it functions by preventing the conversion of testosterone to the more potent androgen dihydrotestosterone. While finasteride has been used in postmenopausal women to treat AGA, finasteride is not approved by the United States Food and Drug Administration (FDA) in women, the primary demographic of patients with CCCA, and is forbidden in pregnant women due to the risk of abnormal sexual development of the fetus [20]. Oral corticosteroids, in short courses, have been suggested to be used in cases with active inflammation [7]; however, they carry a multitude of adverse effects, ranging from mild hypothalamic-pituitary axis suppression to risk of severe infections [21]. In CCCA, systemic corticosteroids may also have the potential for disease relapse upon tapering. Moreover, experts from the Delphi consensus strongly opposed the use of systemic corticosteroids for the management of CCCA [9].

Procedural therapies such as LED light therapy, PRP, and exosomes were not used by any respondents. This may reflect limited clinical evidence of these emerging therapies, lack of provider familiarity or access, and significant cost barriers to the patient, particularly in the context of a patient population that is largely unwilling or unable to pursue out-of-pocket therapies. LED light therapy, specifically low-level light therapy, is hypothesized to have an anti-inflammatory role through its proposed action on the cytochrome complex mitochondrial pathway [22]. A recent case series exploring the use of this therapy in patients with CCCA suggested potential benefit as an adjuvant therapy [23]. PRP, an autologous blood product with high concentrations of platelets, growth factors (GFs), and cytokines, is hypothesized to exert anti-inflammatory effects and stimulate new follicles via growth factors [24]. Exosomes, which are small membrane-bound extracellular vesicles, have an unclear mechanism of action but are proposed to modulate the hair cycle through paracrine signaling by promoting the transition from telogen to anagen and have anti-inflammatory properties [25,26]. However, exosome therapy is not approved by the FDA, which has issued consumer alerts regarding the unregulated nature of exosome products in the United States [27]. To date, evidence supporting the use of these emerging procedural therapies remains limited to a small number of case reports, underscoring the need for further clinical investigation before regular implementation into clinical practice.

Limitations and strengths

This study is primarily limited by its small sample size and the restriction of survey distribution to two dermatology residency programs, which may limit the generalizability of the findings. Additionally, the inclusion of both residents and attending physicians introduces the possibility of response bias, as there is a possibility that trainees may be influenced by the prescribing patterns and preferences of their supervising attendings. The cross-sectional design of the survey also carries the risk of sampling bias. Despite these limitations, this study is strengthened by its high response rate and offers additional perspective on CCCA management, particularly within a high SOC patient population. Our study also contributes to the limited body of literature on CCCA, a condition for which many providers continue to rely on anecdotal experience due to the lack of established, evidence-based guidelines for treatment. Future studies should aim to involve larger, multi-institutional cohorts to more comprehensively understand current CCCA treatment patterns to better inform clinical decision-making and guideline development.

## Conclusions

This study provides valuable insight into treatment practices for CCCA by dermatology residents and attendings at a high-CCCA volume academic institution in the absence of standardized guidelines. While most approaches aligned with the current literature, there were some divergences in clinical practices, such as the use of topical minoxidil as a first-line therapy and a lower utilization of oral doxycycline compared to existing literature. Our data may reflect restricted access to therapies, variability in provider comfort in CCCA management, concern for side effects of systemic treatments, and presentations of later, “burnt-out” stages of CCCA. The results reinforce the need for randomized control trials to establish clinically validated treatment algorithms and improve physician confidence in managing this challenging disease.

## Appendices

Appendix 1 

**Table 2:** CCCA questionnaire CCCA: central centrifugal cicatricial alopecia; PGY: post-graduate year; TAC: triamcinolone; LED: light-emitting diode; PRP: platelet-rich plasma

Demographics:	
1. What race do you identify with most?	a. American Indian or Alaskan Native
	b. Asian
	c. Black
	d. Native Hawaiian or Other Pacific Islander
	e. White
	f. Prefer not to answer
2. What ethnicity do you identify with most?	a. Hispanic
	b. Not-Hispanic
	c. Prefer not to answer
3. What type of location do you primarily practice in?	a. Urban
	b. Suburban
	c. Rural
4. What type of setting do you primarily practice?	a. Solo (private practice)
	b. Group (private practice)
	c. Multispecialty (either academics or private practice)
	d. Academic
	e. Government
5. How long have you been in practice:	a. 1-3 years
	b. 4-9 years
	c. 10-14 years
	d. 15+ years
	e. Currently in training (PGY-1, PGY-2, PGY-3 or PGY-4)* Free response
6. What is the mix of SOC patients seen in your practice?:	a. None
	b. <10%
	c. 25%
	d. 50%
	e. 75%
	f. >75%
7. How many central centrifugal cicatricial alopecia (CCCA) patients do you see per week?:	a. None
	b. <3
	c. 4-10
	d. 11-20
	e. More than 20
8. What percentage of your patients are willing to use out-of-pocket therapies?:	a. None
	b. <10%
	c. 25%
	d. 50%
	e. 75%
	f. >75%
9. How comfortable are you with treating hair loss?	a. Very comfortable (1)
	b. Comfortable (2)
	c. Neutral (3)
	d. Uncomfortable (4)
	e. Very uncomfortable (5)
10. How comfortable are you with treating CCCA?:	a. Very comfortable (1)
	b. Comfortable (2)
	c. Neutral (3)
	d. Uncomfortable (4)
	e. Very uncomfortable (5)
The following questions are about therapies you use in practice:	
1. Do you recommend/prescribe ketoconazole shampoo for CCCA?	a. No
	b. Yes
If yes to Q1:	
1a. How likely are you to recommend/prescribe this treatment (ketoconazole shampoo)?	a. First line (every patient with CCCA)
	b. Second line (after one therapy has been tried)
	c. Third line (after two therapies have been tried)
	d. With certain conditions *Free response
1b. How confident are you in the efficacy of this treatment (ketoconazole shampoo) to treat scaling, itchiness, inflammation, and/or reduce hair loss?	a. Very confident (1)
	b. Confident (2)
	c. Neutral (3)
	d. Not confident (4)
	e. Very not confident (5)
1c. What percentage of this therapy (ketoconazole shampoo) do you recommend/prescribe?	a. 1%
	b. 2%
1d. What frequency of this therapy (ketoconazole shampoo) do you recommend/prescribe?	a. Daily
	b. Three times a week
	c. Once a week
	d. As needed
1e. How often do you counsel patients on the side effects of dryness/brittleness of hair associated with ketoconazole use?	a. Always
	b. Often
	c. Sometimes
	d. Not at all
2. Do you recommend/prescribe ciclopirox shampoo for CCCA?	a. No
	b. Yes
If yes to Q2:	
2a. *How likely are you to recommend/prescribe this treatment (ciclopirox shampoo)?	a. First line (every patient with CCCA)
	b. Second line (after one therapy has been tried)
	c. Third line (after two therapies have been tried)
	d. With certain conditions *Free response
2b. How confident are you in the efficacy of this treatment (ciclopirox shampoo) to treat scaling, itchiness, inflammation, and/or reduce hair loss?	a. Very confident (1)
	b. Confident (2)
	c. Neutral (3)
	d. Not confident (4)
	e. Very not confident (5)
2c. What frequency of this therapy (ciclopirox shampoo) do you recommend/prescribe?	a. Daily
	b. Three times a week
	c. Once a week
	d. As needed
3. Do you recommend/prescribe zinc pyrithione shampoo for CCCA?	a. No
	b. Yes
If yes to Q3:	
3a. *How likely are you to recommend/prescribe this treatment (zinc pyrithione shampoo)?	a. First line (every patient with CCCA)
	b. Second line (after one therapy has been tried)
	c. Third line (after two therapies have been tried)
	d. With certain conditions *Free response
3b. How confident are you in the efficacy of this treatment (zinc pyrithione shampoo) to treat scaling, itchiness, inflammation, and/or reduce hair loss?	a. Very confident (1)
	b. Confident (2)
	c. Neutral (3)
	d. Not confident (4)
	e. Very not confident (5)
3c. What frequency of this therapy (zinc pyrithione shampoo) do you recommend/prescribe?	a. Daily
	b. Three times a week
	c. Once a week
	d. As needed
4. Do you recommend/prescribe selenium sulfide shampoo for CCCA?	a. No
	b. Yes
If yes to Q4:	
4a. How likely are you to recommend/prescribe this treatment (selenium sulfide shampoo)?	a. First line (every patient with CCCA)
	b. Second line (after one therapy has been tried)
	c. Third line (after two therapies have been tried)
	d. With certain conditions *Free response
4b. How confident are you in the efficacy of this treatment (selenium sulfide shampoo) to treat scaling, itchiness, inflammation, and/or reduce hair loss?	a. Very confident (1)
	b. Confident (2)
	c. Neutral (3)
	d. Not confident (4)
	e. Very not confident (5)
4c. What frequency of this therapy (selenium sulfide shampoo) do you recommend/prescribe?	a. Daily
	b. Three times a week
	c. Once a week
	d. As needed
5. Do you recommend/prescribe topical steroids for CCCA?	a. No
	b. Yes
If yes to Q5:	
5a. Which topical steroid do you most commonly use?	a. Clobetasol solution/foam (Clobex)
	b. Fluocinolone oil (Derma-smooth oil)
	c. Fluocinonide solution (Lidex)
	d. All of the above
	e. Other *Free response
5b. How likely are you to recommend/prescribe this treatment (topical steroids)?	a. First line (every patient with CCCA)
	b. Second line (after one therapy has been tried)
	c. Third line (after two therapies have been tried)
	d. With certain conditions *Free response
5c. How confident are you in the efficacy of this treatment (topical steroids) to treat scaling, itchiness, inflammation, and/or reduce hair loss?	a. Very confident (1)
	b. Confident (2)
	c. Neutral (3)
	d. Not confident (4)
	e. Very not confident (5)
5d. What is the typical dosage of this therapy (topical steroids) that you recommend/prescribe?	a. Twice a day
	b. Daily
	c. Three times a week
	d. Once a week
	e. As needed
6. Do you use intralesional steroids (Kenalog or triamcinolone) for CCCA?	a. No
	b. Yes
If yes to Q6:	
6a. How likely are you to recommend/prescribe this treatment?	a. First line (every patient with CCCA)
	b. Second line (after one therapy has been tried)
	c. Third line (after two therapies have been tried)
	d. With certain conditions *Free response
6b. How confident are you in the efficacy of this treatment to treat scaling, itchiness, inflammation, and/or reduce hair loss?	a. Very confident (1)
	b. Confident (2)
	c. Neutral (3)
	d. Not confident (4)
	e. Very not confident (5)
6c. What concentration of this therapy (intralesional steroids) do you use?	a. TAC 5%
	b. TAC 10%
	c. TAC 20%
	d. TAC 40%
	e. Other *Free response
6d. What is the typical dosage of this therapy (intralesional steroids) that you administer per visit?	f. <1mL
	g. 1-3mL
	h. 3-5mL
	i. >5mL
7. Do you use topical minoxidil for CCCA?	a. No
	b. Yes
If yes to Q7:	
7a. How likely are you to recommend/prescribe this treatment (topical minoxidil)?	a. First line (every patient with CCCA)
	b. Second line (after one therapy has been tried)
	c. Third line (after two therapies have been tried)
	d. With certain conditions *Free response
7b. How confident are you in the efficacy of this treatment (topical minoxidil) to treat inflammation, and/or reduce hair loss?	a. Very confident (1)
	b. Confident (2)
	c. Neutral (3)
	d. Not confident (4)
	e. Very not confident (5)
7c. What percentage of this therapy (topical minoxidil) do you recommend/prescribe?	a. 2%
	b. 5%
	c. >5%
	d. Other *Free response
7d. How often do you counsel your patients on the side effects of temporary hair shedding, irritation, and hypertrichosis associated with minoxidil use?	a. Always
	b. Often
	c. Sometimes
	d. Not at all
8. Do you use oral minoxidil for CCCA?	a. No
	b. Yes
If yes to Q8:	
8a. How likely are you to recommend/prescribe this treatment (oral minoxidil)?	a. First line (every patient with CCCA)
	b. Second line (after one therapy has been tried)
	c. Third line (after two therapies have been tried)
	d. With certain conditions *Free response
8b. How confident are you in the efficacy of this treatment (oral minoxidil) to treat inflammation, and/or reduce hair loss?	a. Very confident (1)
	b. Confident (2)
	c. Neutral (3)
	d. Not confident (4)
	e. Very not confident (5)
8c. What is the typical dosage of this therapy that you prescribe?	a. 0.625mg (1/4 pill) daily
	b. 1.25mg (1/2 pill) daily
	c. 2.5mg (full pill) daily
	d. 5mg (2 pills) daily
9. Do you use oral finasteride for CCCA?	a. No
	b. Yes
If yes to Q9:	
9a. How likely are you to recommend/prescribe this treatment (oral finasteride)?	a. First line (every patient with CCCA)
	b. Second line (after one therapy has been tried)
	c. Third line (after two therapies have been tried)
	d. With certain conditions *Free response
9b. How confident are you in the efficacy of this treatment (oral finasteride) to treat inflammation, and/or reduce hair loss?	a. Very confident (1)
	b. Confident (2)
	c. Neutral (3)
	d. Not confident (4)
	e. Very not confident (5)
9c. What is the typical dosage of this therapy (oral finasteride) that you prescribe?	a. 1mg daily
	b. 5mg daily
10. Do you use oral antibiotics (tetracyclines) for CCCA?	a. No
	b. Yes
If yes to Q10:	
10a. How likely are you to recommend/prescribe this treatment (oral antibiotics)?	a. First line (every patient with CCCA)
	b. Second line (after one therapy has been tried)
	c. Third line (after two therapies have been tried)
	d. With certain conditions *Free response
10b. How confident are you in the efficacy of this treatment (oral antibiotics) to treat inflammation, and/or reduce hair loss?	a. Very confident (1)
	b. Confident (2)
	c. Neutral (3)
	d. Not confident (4)
	e. Very not confident (5)
10c. What is the typical dosage of this therapy (oral antibiotics) that you prescribe?	. *Free response
11. Do you use in-office LED light therapy for CCCA?	a. No
	b. Yes
If yes to Q11:	
11a. How likely are you to recommend/prescribe this treatment (LED light therapy)?	a. First line (every patient with CCCA)
	b. Second line (after one therapy has been tried)
	c. Third line (after two therapies have been tried)
	d. With certain conditions *Free response
11b. What device and wavelength do you have in-office?	*Free response
11c. How confident are you in the efficacy of this treatment (LED light therapy) to treat inflammation, and/or reduce hair loss?	a. Very confident (1)
	b. Confident (2)
	c. Neutral (3)
	d. Not confident (4)
	e. Very not confident (5)
11d. How frequently do patients come in for LED light therapy treatments?	a. Three times per week
	b. Once per week
	c. Other *Free response
12. Do you use platelet-rich plasma (PRP) injections for CCCA?	a. No
	b. Yes
If yes to Q12:	
12a.How likely are you to recommend/prescribe this treatment (PRP)?	a. First line (every patient with CCCA)
	b. Second line (after one therapy has been tried)
	c. Third line (after two therapies have been tried)
	d. With certain conditions *Free response
12b. How confident are you in the efficacy of this treatment (PRP) to treat inflammation, and/or reduce hair loss?	a. Very confident (1)
	b. Confident (2)
	c. Neutral (3)
	d. Not confident (4)
	e. Very not confident (5)
12c. What PRP system do you use?	a. ProGen
	b. Eclipse
	c. Emcyte
	d. PRFM- Selphyl
	e. Other *Free response
13. Do you use exosomes for CCCA?	a. No
	b. Yes
If yes to Q13:	
13a. How likely are you to recommend/prescribe this treatment (exosomes)?	a. First line (every patient with CCCA)
	b. Second line (after one therapy has been tried)
	c. Third line (after two therapies have been tried)
	d. With certain conditions *Free response
13b. How confident are you in the efficacy of this treatment (exosomes) to treat inflammation, and/or reduce hair loss?	a. Very confident (1)
	b. Confident (2)
	c. Neutral (3)
	d. Not confident (4)
	e. Very not confident (5)
14. Do you use any compounded topical treatments for CCCA?	a. No
	b. Yes
If yes to Q14:	
14a. If yes, which compounded topical treatments?	a. Clobetasol/minoxidil/tacrolimus
	b. Tacrolimus
	c. Other *Free response
15. Any additional treatments you use for patients with CCCA, please list them here:	*Free response
